# Sugar Intake Elicits Intelligent Searching Behavior in Flies and Honey Bees

**DOI:** 10.3389/fnbeh.2018.00280

**Published:** 2018-11-28

**Authors:** Axel Brockmann, Pallab Basu, Manal Shakeel, Satoshi Murata, Naomi Murashima, Ravi Kumar Boyapati, Nikhil G. Prabhu, Jacob J. Herman, Teiichi Tanimura

**Affiliations:** ^1^National Centre for Biological Sciences, Tata Institute of Fundamental Research, Bengaluru, India; ^2^International Centre for Theoretical Sciences, Tata Institute of Fundamental Research, Bengaluru, India; ^3^The University of Trans-disciplinary Health Sciences and Technology, Bengaluru, India; ^4^Graduate School of Systems Life Sciences, Kyushu University, Fukuoka, Japan; ^5^Department of Integrative Biology, The University of Texas at Austin, Austin, TX, United States

**Keywords:** search behavior, food reward, path integration, landmark learning, dance communication, *Drosophila melanogaster*, *Apis mellifera*

## Abstract

We present a comparison of the sugar-elicited search behavior in *Drosophila melanogaster* and *Apis mellifera*. In both species, intake of sugar-water elicits a complex of searching responses. The most obvious response was an increase in turning frequency. However, we also found that flies and honey bees returned to the location of the sugar drop. They even returned to the food location when we prevented them from using visual and chemosensory cues. Analyses of the recorded trajectories indicated that flies and bees use two mechanisms, a locomotor pattern involving an increased turning frequency and path integration to increase the probability to stay close or even return to the sugar drop location. However, evidence for the use of path integration in honey bees was less clear. In general, walking trajectories of honey bees showed a higher degree of curvature and were more spacious; two characters which likely masked evidence for the use of path integration in our experiments. Visual cues, i.e., a black dot, presented underneath the sugar drop made flies and honey bees stay closer to the starting point of the search. In honey bees, vertical black columns close to the sugar drop increased the probability to visit similar cues in the vicinity. An additional one trial learning experiment suggested that the intake of sugar-water likely has the potential to initiate an associative learning process. Together, our experiments indicate that the sugar-elicited local search is more complex than previously assumed. Most importantly, this local search behavior appeared to exhibit major behavioral capabilities of large-scale navigation. Thus, we propose that sugar-elicited search behavior has the potential to become a fruitful behavioral paradigm to identify neural and molecular mechanisms involved in general mechanisms of navigation.

## Introduction

Food and nest search behaviors are the most successful experimental paradigms to study navigation and spatial memory in insects and vertebrates (Jeffery, 2003; Collett et al., 2013; Webb and Wystrach, 2016). Search for food can be separated into two distinct phases: a hunger induced large-scale search for food sources and a food intake elicited local search for more food (Bell, 1990). Interestingly, 60 years ago the American entomologist Vincent Dethier suggested that a simple sugar-elicited local search behavior observed in solitary flies might represent an ancestral behavioral locomotor pattern that was co-opted during evolution into the honey bee dance behavior which communicates the distance and direction to a food source (Dethier, 1957; Frisch, 1967). Sugar-elicited search and honey bee dance are similar in that they are initiated after the intake of food and include a stereotypic turning behavior; which is modulated by the reward value of the food source and the internal state of the individual (Dethier, 1957; Frisch, 1967; Barron et al., 2007).

Dethier’s ideas have not been taken up by bee researchers. To test whether honey bees show sugar-elicited search behavior and whether this assay could be useful to study molecular or neuronal mechanisms involved in navigation we performed a series of comparative studies with honey bees and fruit flies. We focused on three questions. First, do honey bees actually show sugar-elicited search behavior, second if so, how similar is this search behavior in solitary flies and social honey bees, and third, is the search behavior based on a simple increase in turning frequency or does it involve more complex mechanisms of spatial orientation. For example, Kim and Dickinson (2017) recently provided first evidence that yeast-induced local search behaviors in mated *Drosophila* females involves path integration. We developed similar behavioral assays for flies and bees and then tested different aspects of the search behavior: (a) effect of sugar concentration on the intensity of search behavior, (b) effect of lighting condition on search trajectories, (c) the capability of sugar intake to induce learning processes that might affect the search trajectory.

Our experiments showed that social honey bees initiated a search behavior after ingesting a drop of sugar which is quite similar to that of solitary flies. More importantly, our analyses indicated that sugar-elicited search behavior is not just a simple turning behavior but involves a set of complementary responses: change in turning frequency, path integration and initiation of learning processes. Our findings suggest that this small-scale spatial orientation behavior involves behavioral capabilities and strategies present in large-scale navigation (Frisch, 1967; Wehner and Srinivasan, 2003; Collett et al., 2013; Wehner et al., 2016). Thus, sugar-elicited search behavior promises to be a fruitful behavioral paradigm to study general neural and molecular mechanisms navigation.

## Materials and Methods

### Drosophila melanogaster

Male flies of the Canton-S strain of *Drosophila melanogaster* were used throughout. Male and female flies both show sugar-elicited search behavior. However, we only used male flies because starvation time is more consistent among male flies and female flies change their feeding preference after mating (Murata et al., 2017). Flies were raised on glucose-cornmeal-yeast-wheat germ medium at 25°C, and a 12-h light/dark cycle. Behavioral experiments were done during the central 4 h of the light period. Pilot experiments indicated that the flies showed the highest searching activity during the central 4 h daytime. Flies eclosed within 12 h period were collected and maintained in a fresh medium for 1 day and afterward starved for 24 h in a vial with a water (evian^®^) soaked Kimwipe paper at the bottom.

For the experiments, single flies were transferred into 0.5 ml microcentrifuge tubes. The tube was placed on the LED lighting box for more than 3 min for acclimatization. A Petri-dish was placed on the lighting box and a tiny amount of silicone oil was placed in the center of the Petri-dish to hold the 0.1 μl of 200 mM sucrose solution. The solution was colored with a blue food dye to detect the presence of sugar solution. Then we placed the tube over the sugar droplet and waited until the fly found the droplet. Immediately after the fly started to ingest the solution, we removed the tube and surrounded the Petri-dish with a white cylindrical tube (67 mm inner diameter × 100 mm height, polyvinylchloride resin) and started the video recording (Logicool HD Webcam C615, or NET COWboy DC-NCR20U, Digital Cowboy). Recordings were done for 3 min at 30 f/s.

Experiments under dark conditions were performed in a dark room (<1 lux, measured by CENTER 337 digital mini luxmeter; Center Technology Corp., Taiwan) and the arena was illuminated with infrared LEDs (850 nm, S8100-60-B/C-IR, Scene Electronics, China). Spectrometer (QE65000, Ocean Optics, United States) measurements confirmed that the spectrum ranged from about 730 to 930 nm with a maximum emission at 846 nm. Preparation and positioning of the flies were done under the dim deep red light. When the fly commenced ingestion of the solution and we turned off the light and started the video recording (DC-NCR20U, Digital Cowboy or Flea3, Point Gray (40 f/s, 1214 mm lens, Azure).

We did two sets of experiments to demonstrate that flies are capable of returning to the sugar drop location without visual and chemosensory cues. The first set of experiments was done using light condition (Petri-dish surrounded with a white cylindrical tube, 67 mm inner diameter × 100 mm height, polyvinylchloride resin). The light source consisted of an array of white light LEDs positioned below the arena. The arena was made of opaque milk glass which diffused the light generating an almost homogeneous light distribution. Even if there were slight differences, we did not observe any spatial skew in the walking trajectories. The sugar drop was presented on a removable band (band width 5 mm). The second set was done in dark conditions (see above) and the sugar drop was presented on a transparent disk (small disk: 5.6 mm diameter, large disk: 17 mm diameter). The disks were 0.175 mm in thickness and made of clear polyester sheet. The disk was immediately removed when the fly started walking and left the disk.

For the experiments testing the effect visual cues associated with the sugar drop we used a blue rectangular cellophane film (2 × 2 mm) which was taped to the bottom of the Petri dish.

All experiments belonging to one set were over several days, but the respective test conditions were alternated.

### Apis mellifera

Experiments were done using nectar foragers of *A. mellifera* colonies that were kept on the NCBS campus, Bengaluru. Colonies were provided with pollen and sugar-water (1 M sucrose) at artificial feeders. Foragers were caught with centrifuge tubes when landing on the feeder before they started to collect sugar-water. Then they were brought to the laboratory and placed in a tube on the arena. After 3 min we started the experiment. The sugar drop (3 μl, 2 M sucrose solution) was positioned in the center of the arena (31.5 × 31.5 cm). When the bee started feeding, the tube was removed and the video recording started (40 f/s, Flea3, Point Gray, 1214 mm lens, Azure). Experiments under dark conditions were done in a dark room as described above for *Drosophila* experiments. Spectral sensitivity of the long-wavelength green receptors in *A. mellifera* workers ranges from 330 to 650 nm with a maximum at 544 nm (Peitsch et al., 1992; Chittka and Waser, 1997). In the experiments testing the effect visual cues associated with the sugar drop we used a black dot (diameter: 10 mm) or vertical black cylinders (height: 15 mm; diameter: 6 mm). All experiments stopped when the bees left the arena. After each run the arena was cleaned with 70% ethanol.

### Analyses of Trajectories

Walking trajectories for flies and honey bees were generated using Ctrax software^1^ (Branson et al., 2009). We conceived a new set of MATLAB and Python routines for the analyses of the sugar-elicited search trajectories.

#### Identification of Returns to the Sugar-Drop Location

We developed an algorithm to identify and count the number of returns using two concentric circles. An inner circle indicating the position of the sugar drop (*Drosophila* r0 = 2.5 mm; *A. mellifera* r0 = 7 mm), and the outer circle indicating the minimum distance that a fly or honey bee had to move away from the sugar drop location (*Drosophila*: r1 = 4 mm; *A. mellifera*: r1 = 12 mm). A return was defined as a movement out of the outer circle (r1) and then coming back into the inner circle (r0).

#### Distribution of Return Directions in the Vicinity of the Sugar Drop Location

To analyze whether flies and bees might use non-volatile odors or dispersed minuscule amounts of sugar water for close range orientation we decided to test whether files showed a preferred direction from which they approached the location of the drop. For a given trajectory, we noted all its crossings with the inner and outer circle and saw whether the angles of these crossing are non-randomly distributed. In particular, we considered two nearby virtual circles (*Drosophila* r0 = 5 mm, r1 = 9 mm; *A. mellifera* r0 = 8 mm, r1 = 14 mm), one outer and one inner.

We noted the angles (θ_*i*_) on which the fly (or bee) left the outer circle and reentered the inner circle. We used a statistic inspired by Rayleigh’s Z statistics, defined by,

$$\mathrm{rz}=n\{{\left[\Sigma \cos(\theta_{i})\right]}^{2}+{\left[\Sigma \sin(\theta_{i})\right]}^{2}\}$$

This statistic computes the directionality of a circular distribution. If the directionality is high, that is all the points in a circle are spaced together, then the value of the test statistic is higher. We summed up this statistic for all individual flies (or bees) to get a population level test statistic. The we tested the null hypothesis that the population variance (or spread) of the angular variable θ_*i*_ are less than 30 degrees under the condition of circular normal distribution. 30 degrees roughly corresponds 2 body lengths of flies and bees in the respective video recordings.

We did a bootstrap analysis by generating simulated data assuming our null hypothesis and then used these data to estimate the *p*-value for our experimental data. This analysis was repeated separately for flies and bees and also for light and dark experimental setup. The results were: flies *p* < 1e-05 (Light, Dark); bees *p* < 1e-05 (Light, Dark).

#### Identification of Overlapping Sections in the Search Trajectories

If flies and bees use footprint pheromones to find the way back to the starting position, the individual walking trajectories should show high amounts of overlap, particularly when the insects cannot use vision. We calculated the amount of overlap for fly and bee trajectories that showed sufficient path length. Ctrax generated trajectories have a width of a single pixel (the center of the fly or bee) and they hardly overlap. We considered the actual size of a *Drosophila* male (width: 5–6 pixels) and an *A. mellifera* worker (width: 14–17 pixels) and a selected trajectory width (threshold distance indicating 50% overlap of trajectories: flies = 3 pixels, bees = 7 pixels) to calculate the overlap. As the overlap is meant as a proxy for trail following, we also defined a minimum duration for an overlap (=0.5 s, i.e., 15 frames for flies, and 20 frames for honey bees).

#### Analysis of Return Probabilities

To test whether returns to the sugar drop location were solely due to an increased turning frequency or whether flies and bees had a distinct tendency to return back to the location of the sugar drop, we performed the following analysis on transformed trajectories. First, for each originally recorded trajectory, we defined a new starting point using a virtual circle with a specific radius (*Drosophila*: *r* = 22 mm; *Apis mellifera*:*r* = 45 mm). The crossing point of trajectory and circle was set as the new starting point. The preceding part of the trajectory was discarded. Secondly, to eliminate effects of stopping and grooming behavior, we transformed the trajectories to fixed velocity trajectories, which traverse the same path of the original trajectory but with a fixed velocity. Then, we rotated the generated trajectories so that the new starting point was mapped into a fixed predefined position which allowed to superimpose all transformed trajectories to generate a heat map. We further calculated the marginal probability densities in *x* and *y* directions. The hypothesis was that if the sugar drop location would still show a higher frequency of visitation in the transformed trajectories, turning frequency is not sufficient to explain the returning to the sugar drop observed in the experimental trajectories. We performed this analysis for trajectories under the three different experimental conditions (*Drosophila*: light *n* = 20, dark *n* = 14, disk *n* = 10; *A. mellifera* light *n* = 12, dark *n* = 20, disk *n* = 10). We selected trajectories with multiple returns, because only these would be suitable to test the underlying mechanisms for a higher visitation frequency of the sugar drop location. We performed our analysis with different virtual radii for defining the starting point. However, we observed qualitatively similar behavior in all cases and hence presented our analysis for only the largest radius. To perform a statistical test, we calculated the visitation probability (VP) for a circle with a radius of one insect body length around the original starting point. We defined the VP as the integration of kernel density estimate over the above-mentioned circle. The null hypothesis was that VP could be explained by chance alone. To test this, we performed a bootstrap analysis, in which we randomized the trajectories and calculated the corresponding VP s. A *p*-value is calculated from the number of times the simulated VP s exceeded that of the experiment, divided by the number of simulation runs.

Using trajectories under dark condition, we generated virtual trajectories by randomly re-organizing distance steps. For randomized trajectories, our analysis does not show a distinct tendency to return to the center and we got a probability distribution that is relatively flat and centered around the fictitious point. We presented this analysis for the dark condition, but the results were qualitatively similar for all three experimental conditions.

In the case of the passive displacement experiments, we did the same analysis with the new shifted location as the origin.

## Results

### Sugar-Elicited Search Behavior in Flies and Honey Bees

**FIGURE 1:**
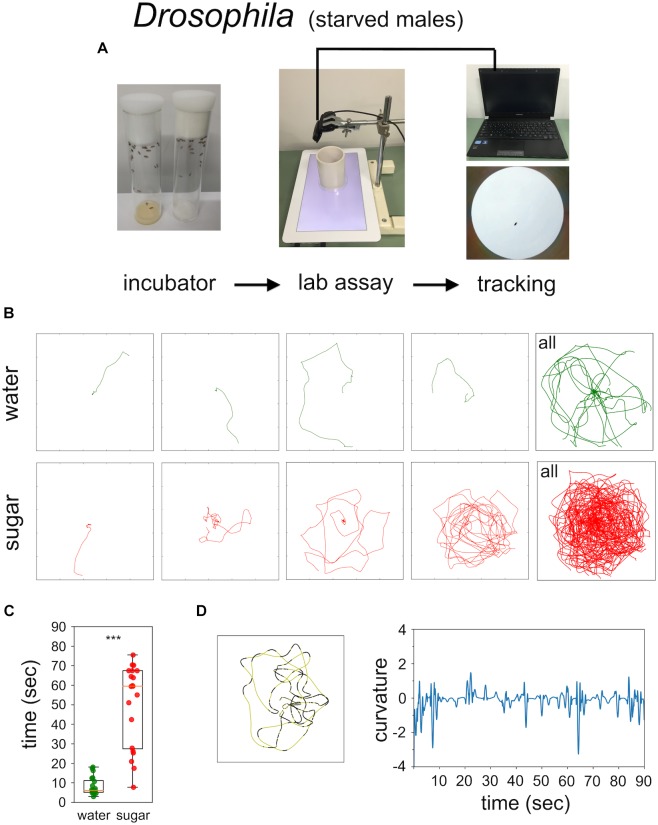
Sugar-elicited search behavior (SeS) in flies. **(A)** Scheme of experimental procedure. **(B)** Selected walking trajectories of individual flies. Green trajectories: water; red trajectories: 0.2 M sugar-water. **(C)** Comparison of stay time – time till leaving the arena – between flies having ingested water (green) or 0.2 M sugar-water (red; Mann–Whitney *U*-test *p* < 0.001). ^∗∗∗^*p* < 0.001. **(D)** Example search run and analysis of curvature. Flies preferentially walked in fairly straight path (yellow sections) interrupted with abrupt large changes in direction (black sections).

**FIGURE 2:**
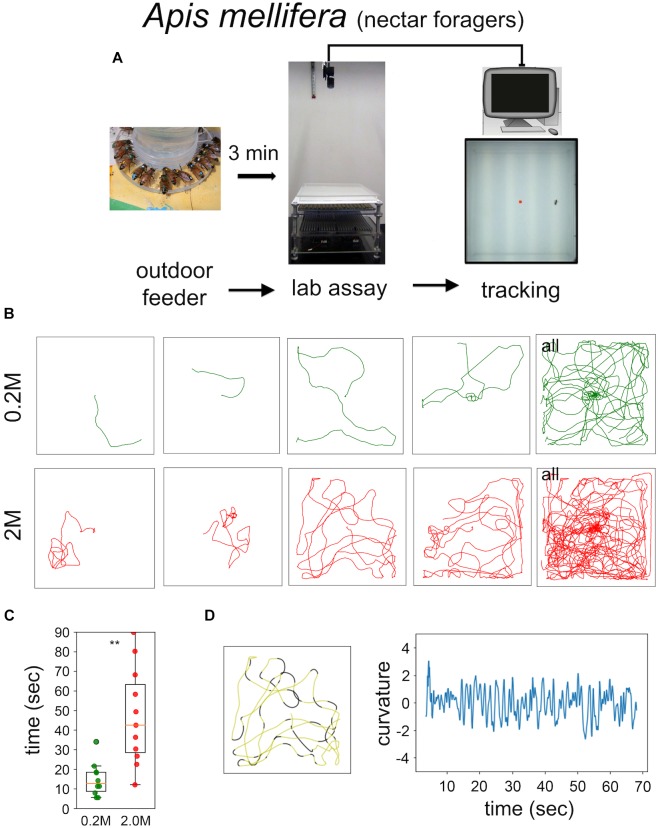
Sugar-elicited search behavior (SeS) in honey bee nectar foragers. **(A)** Scheme of experimental procedures. **(B)** Selected walking trajectories of individual honey bee nectar foragers. Green trajectories: 0.2 M; red trajectories: 2.0 M sugar-water. **(C)** Comparison of stay time – time till leaving the arena – between honey bees having ingested 0.2 (green) or 2.0 M sugar-water (red; Mann–Whitney *U*-test *p* = 0.001). ^∗∗^*p* < 0.01. **(D)** Example search run and analysis of curvature. Honey bees showed a more meandering walking pattern compared to *Drosophila* (yellow sections: straight path; black sections: large changes in directions).

Food-starved flies and highly motivated honey bee nectar foragers were transferred in small vials to the experimental arena. After 3 min of adaptation a single fly, or a single honey bee, respectively, was carefully positioned close to the non-smelling sugar drop so that they started feeding (Figures 1, 2). After the intake of a little drop of water or sugar-solution both insects started a variety of walking responses. The least response was a short relatively straight walking path and a rapid flying off. Most intricate trajectories consisted of initially small circles which increased in size with time (Figures 1, 2). Staying time (i.e., time till leaving the experimental arena significantly differed between water and sugar-water fed flies as well as between low and high concentrated sugar water fed honey bees (Figure 1C Mann–Whitney *U*-test *p* < 0.001; Figure 2C Mann–Whitney *U*-test *p* = 0.001). Flies walked in bouts frequently stopping for a short time. These walking bouts were relatively straight and flies mainly changed directions performing sharp turns during the stops (Figure 1D; see also Martin et al., 1999; Geurten et al., 2014). In all our experiments flies showed a high amount of grooming which is likely due to the relatively large sugar water volume fed (Taylor et al., 2015; Murata et al., 2017). In contrast, honey bees moved for long stretches slightly changing directions of the path (Figure 2D).

### Visual, Olfactory and Gustatory Cues Are Not Necessary to Return to the Sugar Drop Location

**FIGURE 3:**
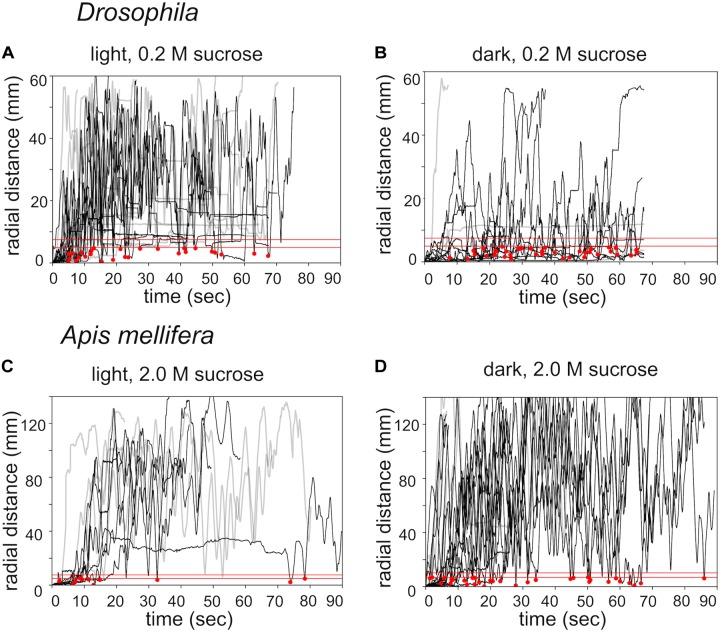
Effects of lighting conditions on search behavior. Time plots of radial distances for search trajectories induced by different food rewards and different lighting conditions. **(A)** *Drosophila*: light condition / 0.2 M sugar-fed *n* = 14. **(B)** *Drosophila*: dark condition / 0.2 M sugar-fed *n* = 12. **(C)** *Apis mellifera*: light condition / 2.0 M sugar-fed *n* = 9. **(D)** *A. mellifera*: dark condition / 2.0 M sugar-fed *n* = 10. Black lines: walking trajectories with returns to location of the sugar drop; gray lines: no returns. Red lines: threshold distances to identify a return to the sugar drop location (see experimental procedures). The upper red line (r1): *Drosophila* r1 = 4 mm, *A. mellifera* r1 = 12 mm); lower red line (r0): *Drosophila* r0 = 2.5 mm, *A. mellifera* r0 = 7 mm); red dots: returns to the location of the sugar drop.

Next, we compared search trajectories performed under light and dark (infrared) conditions (Figure 3). Flies and honey bees initiated search behavior under both conditions. Staying time and walking velocity did not differ between light and dark condition for flies and honey bees. However, flies stayed closer to the location of the sugar drop in the dark condition, measured as time-average radial distance during the whole experiment (light 0.2 M: *n* = 14; dark 0.2 M: *n* = 12; Mann–Whitney *U*-test *p*_(2)_ = 0.018, Figures 3A,B), whereas honey bees walked further away from the sugar drop (light 2.0 M: *n* = 9; dark 2.0 M: *n* = 10; Mann–Whitney *U*-test *p*_(2)_ = 0.037, Figures 3C,D).

**FIGURE 4:**
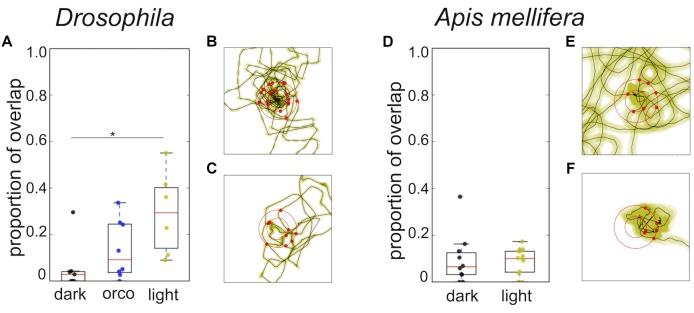
Overlap of trajectories and distribution of return angles. **(A)** *Drosophila*: Comparison of overlap in the trajectories wild-type flies tested in light or dark condition and trajectories of orco mutant flies tested under light condition. Search trajectories showed a significantly higher degree of overlap in light compared to the dark condition (light; *n* = 6; dark; *n* = 7; Mann–Whitney *U*-test *p* = 0.02; Dunn *post hoc* test dmin 3: *p* = 0.022; dmin 5: *p* = 0.035). Orco mutants tested under light conditions showed the tendency to have a higher degree of overlap than wild type flies tested in the dark and a lower degree of overlap than flies tested in light. Yellow: light condition; black: dark condition; blue: *Drosophila* Orco mutant. ^∗^*p* < 0.05. **(B)** *Drosophila*: Example of return directions in one search trajectory in the dark (*n* = 9). **(C)** *Drosophila*: Example of return directions in one search trajectory in light (*n* = 9). Radius of the outer circle: 9 mm. We did not find any statistically significant evidence for a bias in the return directions. **(D)** *A. mellifera:* Search trajectories of honey bees showed a low degree of overlap and did not differ under different light conditions. Yellow: light condition; black: dark condition. **(E)** *A. mellifera*: Example of return directions in one search trajectory in the dark (*n* = 10). **(F)** *A. mellifera*: Example of return directions in one search trajectory in light (*n* = 11). Radius of the outer circle: 14 mm. We did not find any statistically significant evidence for a bias in return directions.

**FIGURE 5:**
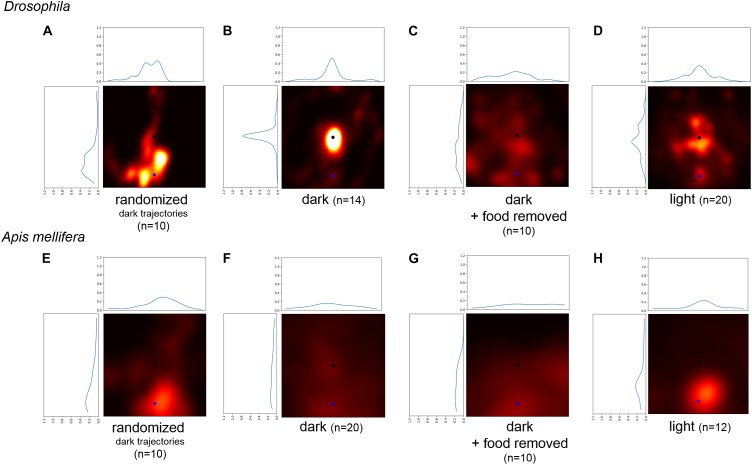
Comparison of the probability to return to a randomly selected point of the trajectory and the location of the sugar-drop for *Drosophila.* **(A)** Probability heatmap of artificial trajectories generated by taking step size and turning angles from real trajectories performed by flies under dark condition and then randomly shuffling the temporal order (*n* = 10). **(B)** Probability heatmap of the spatial distribution of *Drosophila* flies after defining a new virtual starting point at a radial distance of 22 mm using walking trajectories under dark condition (*n* = 14). **(C)** Probability heatmap of the spatial distribution of *Drosophila* flies after defining a new virtual starting point at a radial distance of 22 mm using walking trajectories of the disk experiments under dark condition (*n* = 10). **(D)** Probability heatmap of the spatial distribution of *Drosophila* flies after defining a new virtual starting point at a radial distance of 22 mm using walking trajectories under light condition (*n* = 20). **(E)** Probability heatmap of artificial trajectories generated by taking step size and turning angles from real trajectories performed by honey bees under dark condition and then randomly shuffling the temporal order (*n* = 10). **(F)** Probability heatmap of the spatial distribution of honey bees after defining a new virtual starting point at a radial distance of 45 mm using walking trajectories under dark condition (*n* = 20). **(G)** Probability heatmap of the spatial distribution of honey bees after defining a new virtual starting point at a radial distance of 45 mm using walking trajectories of the disk experiments under dark condition (*n* = 10). **(H)** Probability heatmap of the spatial distribution of honey bees after defining a new virtual starting point at a radial distance of 45 mm using walking trajectories under light condition (*n* = 12).

Closer analysis of the trajectories revealed that flies and honey bees in fact returned to the location of the sugar drop in both conditions (Figure 5, black dots). Thus, we explored whether both species might use chemosensory signals or cues, i.e., footprint pheromones or minuscule amounts of sugar-water or dried sugar crystals (Cederberg, 1977; Ferguson and Free, 1979; Giurfa and Núñez, 1992; Witjes and Eltz, 2009). As a first step to answer this question we tested whether the search trajectories showed any evidence of overlapping trails, which would be present if flies and honey bees marked their walking path. We calculated the amount of overlapping segments for single trajectories (Figure 4). Experiments in which the flies or bees walked only a short distance were excluded from the analysis. Interestingly, *Drosophila*, search trajectories under light condition showed a significantly higher degree of overlap compared to trajectories under dark condition at a line width of 3 pixel (light: *n* = 6; dark: *n* = 7; Mann–Whitney *U*-test *p* = 0.02; Dunn’s *post hoc* test: *p* = 0.022; Figure 4A). In addition, trajectories of the *Orco* mutant with a largely but not totally impaired olfaction (Benton et al., 2009) under light conditions showed a trend to have a higher degree of overlap than trajectories in the dark. Both results suggest that flies predominantly used vision instead of olfaction when they followed their own footprints (Figure 4A). So, what do flies see when they follow their walking paths? One hypothesis is that flies are passively depositing little wax droplets (cuticular hydrocarbons) from their tarsal pads during walking and that they are able to see these droplets or at least light reflections generated by them. The search trajectories of honey bees generally showed a very low degree of overlap independent of the lighting condition (Figure 4C).

To exclude the possibility that flies and honey bees might still use non-volatile contact-chemosensory signals or cues (minuscule droplets of sugar) close to the sugar drop location, we examined if the trajectories showed any sign of preferred directions to approach the sugar drop position. We tested whether in a single search run all the crossing points with a virtual circle around the sugar drop (*Drosophila*: 6 mm; *A. mellifera*: 11 mm) were randomly distributed (Figures 4B,D). In flies as well as in bees, we failed to see a significant directedness in the distribution of return angles irrespective of the lighting conditions (Figures 4B,D; flies (light, dark) *p* < 1e-05; bees (light, dark) *p* < 1e-05).

Based on our results, we propose that sugar-elicited search behavior in flies and bees does not involve trail marking. Furthermore, returns to the sugar drop location are hardly affected by close range low volatile chemosensory cues. Trail marking likely conflicts with the transitory duration of the local search as well as the goal to search in different directions (Bell, 1990). However, it seems that *Drosophila*, and to some lesser degree honey bees, can and do follow their own trails using vision, but it appears that they do not systematically use them to find their way back, because location and direction of overlaps were arbitrarily distributed over the whole trajectory.

### Flies Can Use Self-Motion Information to Return to the Location of the Sugar Drop

There are two possible mechanisms which flies and bees might use to return to the location of the food source without using any environmental cues: (a) the turning behavior increases the probability to return to the starting position of the search trajectory (Wehner and Srinivasan, 1981), or (b) flies and bees use self-motion (idiothetic) information and path integration to intentionally return to the location of the food source (Mittelstaedt and Mittelstaedt, 1980, 2001; Seyfarth et al., 1982; Chittka et al., 1999; Thiélin-Bescond and Beugnon, 2005; Zeil et al., 2013). To decide between these two mechanisms, we generated transformed trajectories of the recorded search runs and then compared the probabilities of returning to the new virtual starting position and the original location of the sugar drop (Figures 5A–H, blue: virtual starting point, black location of the sugar drop). A distinct tendency to return near the vicinity of the original sugar drop location would indicate that returning cannot be explained solely by a higher turning frequency. Trajectories generated by only changing the starting location of the original search runs showed a clear tendency to return to the original sugar drop location in flies (light and dark condition: Bootstrap *p* < 0.0001; dark + food removed: Bootstrap *p* < 0.01). For honey bees, the analysis revealed a distribution with generally higher probabilities for the area around the virtual starting position. Still, the trajectories for the dark and dark + food removed conditions showed a slight tendency to return to the original sugar-drop location (dark condition: Bootstrap p ∼ 0.1; dark + food removed: Bootstrap p ∼ 0.1; Figures 5B,F). Trajectories generated by randomized aligning sections of the experimental search runs (Figures 5A,E) did not show returns to the original sugar drop location. Both findings indicate that characteristics and frequencies of the locomotor pattern during search do not play a role in the returning behavior.

We conclude that our analysis demonstrates that flies use self-motion information (i.e., path integration) during sugar-elicited local search behavior (see also Kim and Dickinson, 2017). The location of the sugar-drop likely functions as a reference point to organize a meaningful search around this location (Wehner and Srinivasan, 1981). For honey bees, only the trajectories under dark condition showed a slight tendency to return to the location of the sugar drop. In general, the walking trajectories of honey bees showed a lesser degree of returning and a greater degree of diffusion compared to flies.

### Passive Displacement Experiment Indicates Path Integration

**FIGURE 6:**
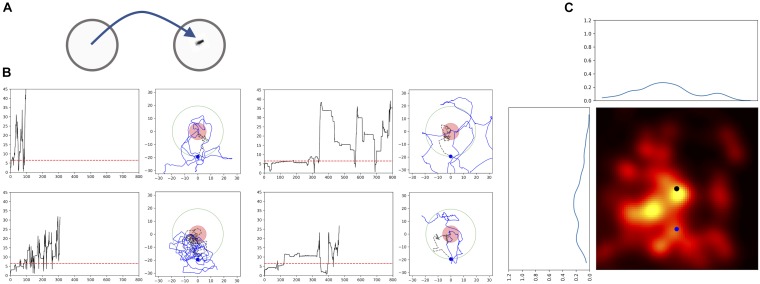
Passive Displacement experiment: walking trajectories of flies that were transferred to a new Petri dish. **(A)** Scheme of the experimental procedure. Flies fed a sugar drop on a plate, and were transferred to a second plate after about 40 s, when they still were feeding. **(B)** Time plots of radial distances and path trajectories of four flies; red dashed line: distance of 2 fly lengths; red circle: circle with radius of 2 fly-length around the position of the fly after transfer; black dashed line: trajectory till crossing the arbitrarily selected circle with a radius of 22 mm; blue dot newly defined virtual starting position for the heat map. **(C)** Probability heatmap of the spatial distribution of *Drosophila* flies after defining a new virtual starting point at a radial distance of 22 mm using walking trajectories. Black dot: original starting position (*n* = 9).

Passive displacement experiments in which the animal is transferred to a new environment are considered to be the most powerful proof that the animals use path integration. Thus, we performed an experiment in which the fly was first allowed to feed on a drop of sugar in one Petri dish and then we transferred it to a new Petri dish (Figure 6A). In most of the experiments (*n* = 9) the flies started a search and showed returns back to the starting point (Figures 6B,C). Analyses using generated transformed trajectories (Figures 6B,C) showed that the returning to the starting point cannot be explained by just increased turning rate alone (Bootstrap *p* < 0.005). Interestingly, these results indicate that the sugar drop location is not necessarily used as the starting point of the search. It appears that the animal itself starts the search and uses the location where it starts the search as the reference point. This is point is usually close to the location of the sugar-water drop, except in the case of the displacement experiment.

### Response to Visual Cues in the Vicinity of the Sugar Drop

**FIGURE 7:**
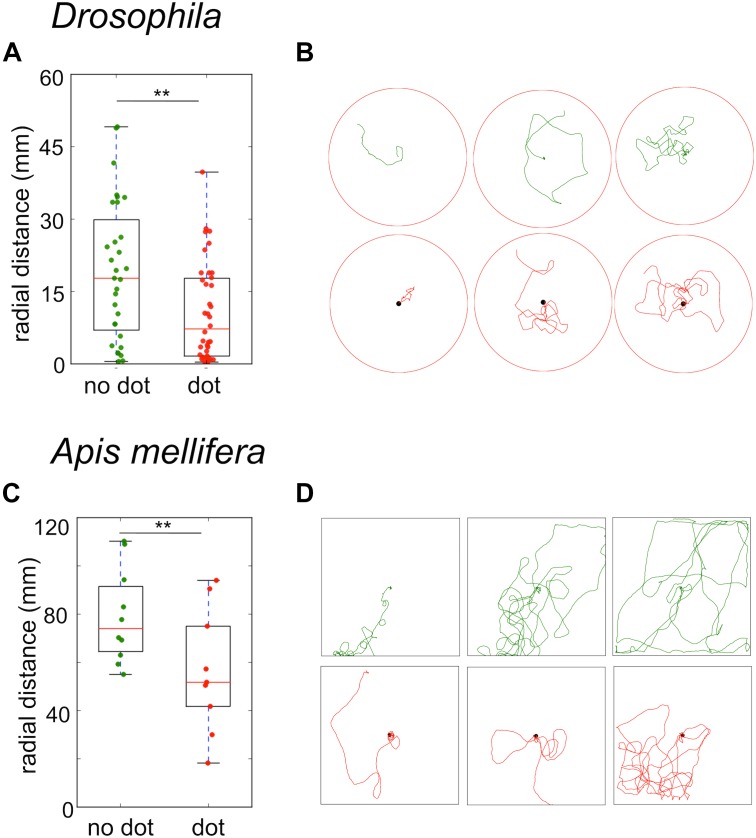
Sugar-elicited search trajectories in the presence of a black dot beneath the sugar reward. **(A)** *Drosophila*: A black dot beneath the sugar drop led to a significant reduction in the mean radial distance of search trajectories. (no dot: median 13 ± 8 mm; black dot: median 5.5 ± 4 mm; Mann–Whitney *U*-test *p*_(2)_ = 0.013). **(B)** *Drosophila*: Examples of sugar-elicited search trajectories of flies without (green) and with a black dot (red) beneath the sugar reward. **(C)** *A. mellifera*: A black dot beneath the sugar drop led to a significant reduction in the mean radial distance in bees (no dot: median 63 ± 11 mm; black dot: 44 ± 18 mm; Mann–Whitney *U*-test *p*_(2)_ = 0.045). ^∗∗^*p* < 0.01. **(D)** *A. mellifera*: Examples of sugar-elicited search trajectories of honey bee nectar foragers without (green) and with a black dot (red) beneath the sugar reward.

Even if flies and bees do not need visual cues to return to the food source, still they should be used to modulate the search trajectory. To test the effect of visual cues on the search behavior, we first presented flies and bees with a black dot beneath the sugar drop. In both species, the strongest effect of the black dot was a significant reduction in the time-averaged radial distance of the trajectories (*Drosophila* no dot: median 13 mm ± 8 mm; black dot: median 5.5 ± 4 mm; Mann–Whitney *U*-test *p* = 0.013; *A. mellifera* no dot: median 63 ± 11 mm; black dot: 44 ± 18 mm; Mann–Whitney *U*-test *p* = 0.045, Figures 7A–D).

**FIGURE 8:**
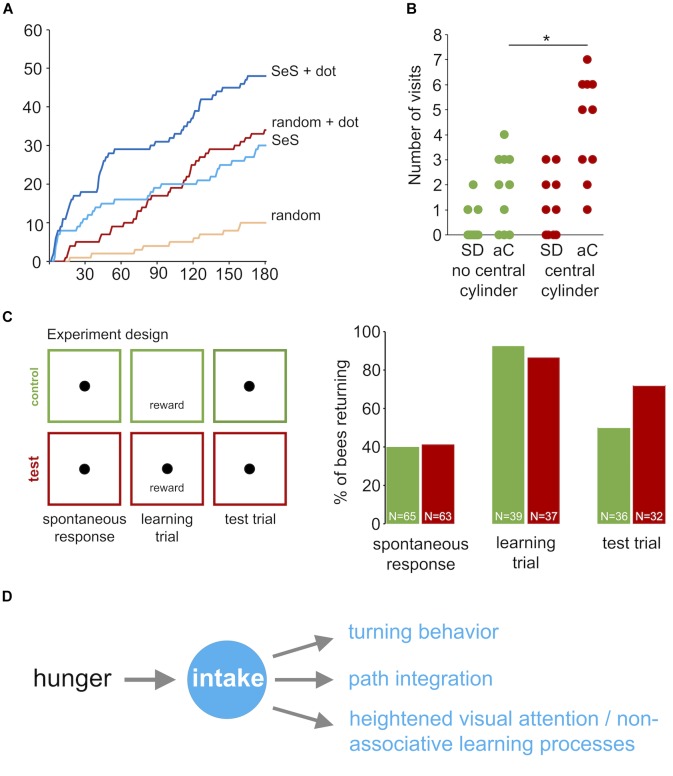
Learning during sugar-elicited search in honey bees. **(A)** Cumulative return number to the location of the sugar-drop during sugar-elicited search behavior and random walk conditions. Orange: random walk without black dot (*n* = 10); red: random walk with black dot (*n* = 10); light blue: sugar-elicited search behavior without black dot (*n* = 10); dark blue: sugar-elicited search behavior with black dot (*n* = 10). A black dot in the center was highly attractive for honey bees exploring the arena without a sugar stimulation (RW compared with RW + dot, Mann–Whitney *U*-test *p* < 0.0001). Further, a black dot did also increase the number of returns in the sugar-elicited search assay (SeS compared with SeS + dot, Mann–Whitney *U*-test *p* < 0.0001). Return frequencies for SeS without dot and random walk with dot over the whole experiment did not significantly differ. However, return frequencies during the SES were initially higher and later in the experiment lower than in the random walk with dot. **(B)** Number of visits to peripherally located black cylinders after honey bee workers encountered or did not encountered a similar black cylinder in the vicinity of the sugar drop. Green: experiment without a cylinder next to the sugar drop (*n* = 10); red: experiment with a cylinder next to the sugar drop (*n* = 10; student *t*-test *p*_(2)_ = 0.004). ^∗^*p* < 0.05. SD: sugar drop; aC: additional cylinders. **(C)** Associative learning experiment. Individual bees were tested in three consecutive trials (3 min, intertrial interval = 3 min). Trial 1: spontaneous return response toward an unrewarded black dot. Then spontaneously responding bees were excluded from trial 2 and 3. Trial 2: Presentation of a sugar drop and induction of sugar-elicited search. Control group: no black dot; test group: black dot underneath the sugar drop. Trial 3: Test run, presentation of an unrewarded black dot. The learning trial changed the proportion of individuals responding to the black dot without any reward (spontaneous response trial: 43.3% (*n* = 60); test trial 73.5% (*n* = 43); Pearson chi-square *p* < 0.005). Percentage of responding bees in the test trial were not significantly different between the control and test group (control group 54.1 % (*n* = 34); test group 73.5% (*n* = 34); Pearson chi-square *p* = 0.08858). **(D)** Scheme: Behavioral responses involved in sugar-elicited search.

To explore the effects of visual cues in the vicinity of the sugar reward in more detail, we performed three additional experiments with honey bees using a closed arena, which allowed studying behavioral responses for a longer time (3 min instead of 90 s). In the first experiment we compared the cumulative number of returns for random walk and sugar-elicited search behavior with and without a black dot indicating the position of the sugar drop (Figure 8A). A black dot in the center of the arena was highly attractive for honey bees exploring the arena without a sugar stimulation (RW compared with RW + dot, Mann–Whitney *U*-test *p* < 0.0001). Further, a black dot also increased the number of returns in the sugar-elicited search assay, particularly in the initial part of the search (SeS compared with SeS + dot, Mann–Whitney *U*-test *p* < 0.0001). Both findings indicated that highly motivated honey bee nectar foragers show a heightened response or attention toward black dots or more precisely high contrast visual cues during general food search and sugar-elicited food search.

**FIGURE 9:**
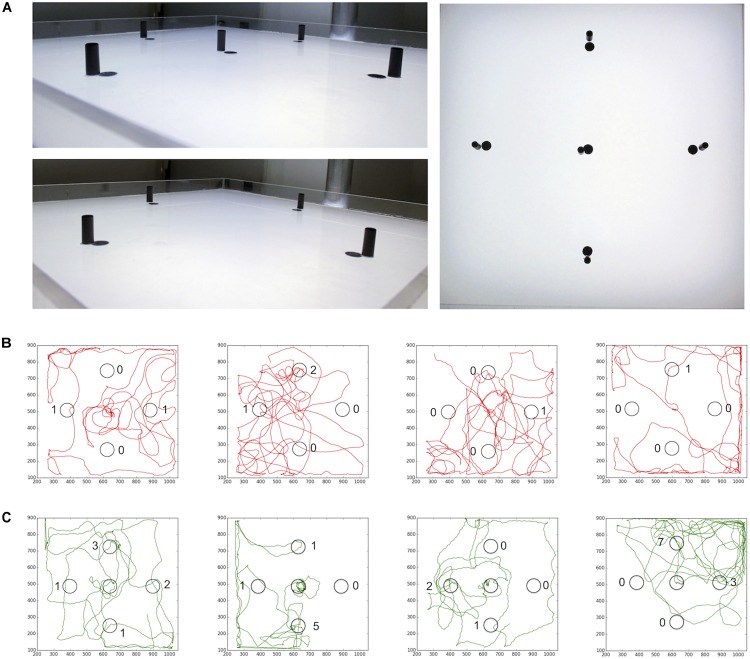
**(A)** Arena for landmark experiments. After the honey bee started to feed from the sugar drop a lid was put over the arena. **(B)** Path trajectories for honey bees experience no landmark close to the sugar drop (red) and **(C)** bees experiences a landmark at the sugar drop location (green).

Then, we asked whether the experience of a black cylinder (height: 15 mm; diameter: 6 mm) close to the sugar drop affects the number of visits to similar additional cylinders in the arena (Figure 9). We used cylinders instead of dots in this experiment to ensure that the bees will see these visual cues from a distance. Honey bees that encountered a black cylinder next to the sugar drop visited distant landmarks significantly more often than those did not encounter a landmark close to the sugar drop (no central cylinder; *n* = 10; central cylinder; *n* = 10; Student’s *t-*test *p*_(2)_ = 0.004; Figure 8B). Thus, the combination of a reward and a visual cue had the capacity to heightened behavioral responses and attention toward similar cues in the vicinity.

Finally, we asked more specifically whether the intake of the sugar drop is capable of inducing an associative learning process. Two groups of bees (test and control group) were exposed to three consecutive experimental runs (trial duration 3 min; intertrial interval 3 min; Figure 8C): (1. trial) A spontaneous response trial, in which bees were released over a black dot without a sugar reward (test group: *n* = 63; control group: *n* = 65). (2. trial) A learning trial, in which the test group of bees was presented the black dot and a drop of sugar water (reward) and the control group of bees were presented a drop of sugar water but no black dot. This trial was only performed with bees that did not respond to the black dot in the first trial (test group: *n* = 37; control group: *n* = 39). Finally, we performed (3. trial) a test trial, in which the bees of the two groups were again presented with the black dot but no reward. Only bees, that showed at least one return in the learning trial were included in this trial (test: *n* = 32; control: *n* = 36). We had two hypotheses. (a) An increase in the percentage of responders between the first trial (spontaneous response) and the third trial would indicate an effect of a sugar intake on responses to visual cues, and (b) a higher percentage of responders in the test group compared to the control group in the third trial would demonstrate that the intake of sugar initiates an associative learning process. The percentage of bees responding in the third trial to the black dot was significantly higher than that in the first trial for the test group (1. trial: 41.3%; 3. trial 71.9%; Pearson chi-square *p* < 0.005) but not for the control group (1. trial: 40%; 3. trial 50%; Pearson chi-square). However, the frequencies of responders between test group and control group in third trial did not differ significantly (test group: 71.9%, control group: 50 %; Pearson chi-square *p* = 0.066; Fischer Exact Probability test one-tailed *p* = 0.055).

## Discussion

The significant finding of our experiments is that sugar-elicited search behavior, first demonstrated by Vincent Dethier, is more intelligent than previously proposed and comprises a set of complementary behavioral responses including an increase in turning frequency, path integration and a capability to initiate learning processes (Figure 8D). Thus, this local search behavior involves behavioral responses and strategies that play major roles in large-scale insect navigation (Frisch, 1967; Jeffery, 2003; Wehner and Srinivasan, 2003; Collett et al., 2013). The basic organization of the sugar-elicited search behavior appeared to be conserved, but flies and honey bees also showed differences, which, for example, might be due to differences in general walking patterns and sensory response thresholds.

White et al. (1984) were the first to suggest that sugar-elicited search behavior involves active returning to the location of the sugar drop. Reasons to return to the original location might be the probability that the food source is not depleted or has been replenished. Flies and bees likely use several different sensory systems, e.g., vision, olfaction, and gustation, to find their way back. However, our removable disc experiments under dark conditions demonstrate that they are capable of repeatedly returning to the start of the search in the absence of visual, olfactory, and gustatory cues. Furthermore, probabilistic analyses using transformed trajectories showed that returning to the sugar drop location cannot be explained solely by an increased turning frequency. Thus, we conclude that flies and likely also bees are capable of using self-motion (idiothetic) cues, e.g., proprioceptive input, to navigate back to the sugar location. The most basic definition of path integration is keeping track of one’s own movement using self-generated (idiothetic) motion signals to be able to return back to the starting point of that movement irrespective of the distances traveled (Seyfarth et al., 1982; Mittelstaedt and Mittelstaedt, 2001). Most recently, Kim and Dickinson (2017) showed that amino-acid starved mated *Drosophila* females after feeding on yeast, started a search behavior similar to the Dethier’s sugar-elicited search behavior that involves path integration. In addition, Zeil et al. (2013) provided some evidence that the Banded Sugar Ant (*Camponotus consobrinus*) likely uses path integration during local search behavior supporting our finding that path integration can be used during small-scale search behaviors.

Our behavioral experiments with honey bees using closed arenas showed that foraging-motivated honey bee foragers are attracted to small black cues or in general contrast differences. Furthermore, intake of the sugar drop showed a tendency to increase the response toward visually obvious vertical columns in the proximity of the sugar-drop location. Adding a column at the location of the sugar drop led to significantly higher number of visits to other vertical columns in the arena compared to the condition in which the sugar-drop was presented without a visual cue. An additional more specific experiment to test for associative learning failed to provide clear statistical evidence, but was suggestive that the intake of sugar-water that initiates the search behavior also has the potential to initiate an associative learning process.

In addition, one should note that in most cases associative learning is initiated after one trial but a significant increase in performance is found only after repeated learning trials (Menzel, 1999; Müller, 2013). Fukushi (1985, 1989) used the sugar-elicited search assay to test color learning in the blow fly *Lucilia cuprina*; and significant effects of associative learning occurred when the sugar-elicited search trial was repeated for several times.

To summarize, the sugar-elicited search behavior in flies and honey bees involves more complex navigational procedures than previously assumed. Although the behaviors are quite similar, flies and bees likely differ in some aspects. For example, the failure to clearly demonstrate the use of path integration in honey bees might not be due to a lack of this capability, but more likely a consequence of a difference in the basic walking strategy which involves more circling and larger-scale walking bouts. On the other hand, learning experiments are easier to perform with honey bees as compared to *Drosophila*. Given our results, Dethier (1957) original suggestion that sugar-elicited search behavior and honey bee dance communication are closely related might not be so wrong (Brockmann and Robinson, 2007; Barron and Plath, 2017). In this case, sugar-elicited search assay provides the opportunity to use *Drosophila* and its neurogenetic toolkit to study the neural circuits and genetic mechanisms underlying food search behavior, navigation, and path integration (Neuser et al., 2008; Sitaraman et al., 2008; Ofstad et al., 2011; Murata et al., 2017). Parallel experiments in honey bees will allow to determine the behavioral differences between flies and a master of insect navigation, as well as verify whether the behavioral responses in the lab assay correspond to those used in large scale navigation in nature (Wehner, 1999; Riley et al., 2003; Jacobs and Menzel, 2014).

## Author Contributions

AB and TT designed the study. MS, SM, NM, RB, and JJH performed the experiments. PB, NP, AB, and TT analyzed the data. AB, PB, and TT wrote the manuscript.

## Conflict of Interest Statement

The authors declare that the research was conducted in the absence of any commercial or financial relationships that could be construed as a potential conflict of interest. The reviewer AW and handling Editor declared their shared affiliation at the time of the review.

## Footnotes

1 http://ctrax.sourceforge.net/
